# A frame-critical policy analysis of Canada’s response to the World Food Summit 1998–2008

**DOI:** 10.1186/2049-3258-72-41

**Published:** 2014-11-24

**Authors:** Catherine L Mah, Catherine Hamill, Krista Rondeau, Lynn McIntyre

**Affiliations:** Division of Community Health and Humanities, Faculty of Medicine, Memorial University, St. John’s, Canada; Dalla Lana School of Public Health, University of Toronto, Toronto, Canada; Department of Community Health Sciences, Faculty of Medicine, University of Calgary, Calgary, Canada; Faculty of Law, McGill University, Montreal, Canada; Institute for Public Health, University of Calgary, Calgary, Canada

**Keywords:** Framing analysis, Public policy, Food insecurity, World food summit

## Abstract

**Background:**

The 2012 visit to Canada of Olivier De Schutter, the United Nations Special Rapporteur on the Right to Food, led to a public rebuff by Canadian governmental officials. This paper adapts the frame-critical policy analysis of Schön and Rein (1994), to explore the rhetorical basis for this conflict. This examination is offered as an illustrative example of how food insecurity is framed as a public policy problem in a high-income nation and how this framing has changed over time.

**Methods:**

We analyze Canada’s decade of sequential responses to the 1996 World Food Summit, spanning 1998–2008, in the form of Canada’s Action Plan on Food Security, and its subsequent Progress Reports. We conducted a qualitative policy analysis, adapting the frame-critical approach first delineated by Schön and Rein (1994). This analysis uses a social constructionist approach to map out the relationships between tacit understanding of policy by particular actors, explicit rhetoric in the public domain, and action in this policy area over time.

**Results:**

We identify three key ways in which competing rhetorical frames arise over time: frame shifts (e.g., a shift away from language highlighting the right to food and health); frame blending (e.g., discussion about poverty becomes obscured by complexity discourse); and within-frame incongruence (e.g., monitoring for health indicators that are unrelated to policy solutions). Together, these frames illustrate how the conflict embodied in the UN Special Rapporteur’s visit has been deeply woven into the policy discourse on food insecurity in Canada over time.

**Conclusion:**

Frame-critical analysis is instructive for exposing and also predicting tensions that impede forward progress on difficult policy issues. Accordingly, such analyses may be helpful in not only dissecting *how* policy can become ‘stuck’ in the process of change but in active reframing towards new policy solutions.

## Background

### Introduction

When asked about food insecurity at the close of his recent mission visit to Canada, Olivier De Schutter, the second United Nations (UN) Special Rapporteur on the Right to Food, remarked, “frankly the question of hunger is not a technical question, it’s a political question” [1]. Special Rapporteurs are independent experts appointed to report and advise on human rights issues by the UN Human Rights Council. De Schutter’s mission to Canada was the first visit by a Special Rapporteur on the Right to Food to a high-income nation. His presence and statements were subsequently disparaged by federal government representatives, who had initially refused meetings at the Ministerial level. The Minister of Health claimed in a press release that she was “surprised that this organization [the UN] is focused on what appears to be a political agenda rather than on addressing food shortages in the developing world… Canada ranks sixth best of all the world’s countries on [the UN] human development index” [2]. This seemed to be a departure from the language in Canada’s *Action Plan on Food Security*, which had expressed that ([3], p10):

In every country, regardless of its wealth or level of poverty, people can be food insecure.

Despite immense differences in per capita incomes, standards of living, resource endowments and many other characteristics which separate countries… many of the same basic dynamics are at work to create food insecurity. In examining Canada's Action Plan, it becomes apparent that there are important parallels between Canada's domestic and international food security concerns, although strategies to resolve them may vary between countries and regions.

In this paper, we ask: What can we learn from the policy language of the UN Special Rapporteur’s public rebuff? Could this conflict have been predicted and characterized based on what we know about food insecurity as a policy problem in Canada?

### Food insecurity in Canada

Food insecurity at the household level, defined as the inability to access sufficient food through socially acceptable means due to income constraints, is a significant public health problem, and a social determinant of health [4, 5]. Food insecurity continues to be a major challenge for populations—albeit within different contexts and based on somewhat different sets of causal factors—in low-, middle-, and high-income nation contexts. Canada is a high-income nation that faces a substantial food insecurity problem. Prior to the last decade, attempts to quantify the prevalence of food insecurity in Canada used inconsistent measures. Beginning in 2004, Canada adopted a systematic measurement of food insecurity by incorporating the Household Food Security Survey Module (HFSSM) in its nationally representative Canadian Community Health Survey (CCHS) [6]. In an analysis of the most recent CCHS data, Tarasuk and colleagues documented a national prevalence of 1.7 million households (12.7%), or approximately 1 in 8, experiencing food insecurity in 2012 [7]. This figure has increased steadily from 11.3% in 2008. This indicator encompasses 4 million individuals, including 1.15 million children, who live in food insecure households across the country. Regional prevalences are exceedingly high; for example 45.2% in the northern territory of Nunavut in 2012.

The 2008 annual report of Canada’s Chief Public Health Officer highlighted food insecurity as an important ongoing public health challenge and reiterated Canada’s commitment to the World Food Summit ([4], p41). Public health actors have been key players in food insecurity policy in Canada, but have also struggled with how to incorporate food insecurity interventions within core public health and health promotion mandates [8]. Whereas other high-income countries comparable to Canada, such as the United States, have adopted national-level policies to address food insecurity, including food assistance programs such as the Supplemental Nutrition Assistance Program, interventions to address food insecurity in Canada have included a patchwork of social policy instruments, public health interventions, and extra-governmental, particularly community-based, action [9].

In this paper, we examine the public tensions that emerged during De Schutter’s visit by reflecting on existing conflicts that are documented as part of the policy discourse on food insecurity in Canada over time. We do so by adapting the frame-critical policy analysis first described by Schön and Rein [10]. This type of analysis uses a social constructionist approach to map out the relationships between tacit understanding of policy by particular actors, explicit rhetoric in the public domain, and policy actions. In our examination, we analyze Canada’s decade of sequential responses to the 1996 World Food Summit, in the form of Canada’s Action Plan on Food Security and its subsequent Progress Reports spanning 1998–2008. This analysis offers an illustrative example of how food insecurity is framed as a public policy problem in a high-income nation and how this framing has changed over time.

## Methods

In this paper, we adapt a qualitative policy analysis approach known as frame-critical analysis, first described by Schön and Rein [10]. Framing theory has been applied in multiple fields of social inquiry over the last few decades, including communications studies, sociology, and political science. Frame-critical policy analyses of interest to readers in public health include recent examinations of alcohol policy in the UK [11]; congestion tax policy in Sweden [12]; and mental health policy in Scotland [13].

Framing analysis focuses on the process of problem definition, an essential element of agenda-setting in public policymaking. Models of agenda-setting describe how policy actors sometimes attempt to “solve problems,” but more often, the process to define what constitutes a policy problem and potential solutions happens independently and can be governed by different social dynamics [14]. Framing analysis can help us to understand how the “puzzle” of household food insecurity can be transformed into “actionable problems” through an analysis of how policy problems are socially constructed ([15], p26). Schön and Rein, whose work remains among the enduring accounts of framing in the policy sciences, interrogate how issues are problematized through conflict and negotiation, which can be analyzed in the rhetoric, or persuasive language, of a policy debate [10, 16, 17]. By asking how household food insecurity is “framed as a policy issue,” we refer to how actors communicate about household food insecurity in terms of its definitions, causes, consequences, who should participate in deciding upon appropriate interventions, why intervention would be necessary or not, and if so, what type of intervention would be appropriate and effective. In other words, a frame is language that conveys an underlying causal story about a policy problem and what should be done about it—a diagnosis and a prescription [18]. How policy actors describe their answers to these questions is more important than what they would prescribe.

Frame-critical analysis attempts to make the tacit elements of policy conflicts explicit by identifying the issue terrain; naming competing frames within the debate; and positing the dynamics of those frames in action – e.g., how reframing has occurred over time [18]. This type of analysis has two functions. It permits hypothesis-generation, and consequently empirical analysis of other policy data for hypothesis-testing ([10], p36). It also has a normative dimension: it is intended to be a first step towards resolution of persistent or intractable conflicts.

In this paper, we have purposively selected Canada’s World Food Summit reports, including its *Action Plan for Food Security*[3] and the five subsequent *Progress Reports*[19–23], as an important document set for initiating an analysis on how food insecurity has been defined and framed over time in Canada. In addition to the institutional sanction of the reports at the national level, a broad array of interventions are embedded within the *Action Plan* and *Progress Reports* under the food insecurity banner, which makes them useful for considering how public health interventions are situated within the national health and social policy context.

We conducted the analysis as follows. First, we contextualized each of the documents, indexing meta-information on the structure, authorship, and format of the reports. Second, we carried out an extraction of texts focusing on domestic policy instruments, particularly social policy, to address food insecurity. Extracts focusing solely on agricultural production were excluded. Text extracts were qualitatively coded by one individual; emergent themes and patterns were peer-debriefed on an ongoing basis by all members of the research team. This allowed us to produce a *post-hoc* classification of major programs and policy actions to address food insecurity according to specific needs, such as education, capacity building, income support, food, food distribution, and so on. We then analyzed the findings across time and situated them within Canadian political environment to identify the dynamics of frames in action, encompassing three key patterns: frame shifts, frame dilution, and within-frame conflict.

## Results

### Examining Canada’s commitment to the World Food Summit

The 1996 World Food Summit (WFS) was hosted by the Food and Agriculture Organization (FAO) in Rome and was attended by 112 state leaders and over 70 other high-level representatives. Its imperative was to ‘eradicate’ hunger and poverty, and it defined food *security* as existing when “all people, at all times, have physical, social and economic access to sufficient safe and nutritious food that meets their dietary needs and food preferences for an active and healthy life” [24]. Note that in this paper we focus on food insecurity, which specifies lack of access to food because of financial constraint [25]. The collective nation-state commitment to the WFS was recorded in the *Rome Declaration on World Food Security* and the *World Food Summit Plan of Action*, which described a pathway to addressing food insecurity within and across countries [24].

The specific role of Canada in the WFS process and related international food and nutrition developments of the early 1990s has been well described elsewhere [26, 27]. It is worth reiterating that Canada was a key player at the Summit, including drafting of language and helping to convene a non-governmental organization side forum. The Canadian federal agriculture department, Agriculture and Agri-Food Canada, was responsible for coordinating the contribution to the WFS, as well as issuing the domestic response and progress reports.

After the Summit, Agriculture and Agri-Food Canada assembled a 37-member Joint Consultative Group to articulate Canada’s approach to WFS pledges through Canada’s *Action Plan for Food Security*[3], featuring inter-ministerial representation as well as civil society actors from health, agriculture, natural resources, and development sectors. The *Action Plan* identified seven central commitments and ten priorities for action. The seven Canadian commitments, derived from WFS commitments, describe domestic and international actions:

1. *An Enabling Environment*, defined in terms of social, political, and economic factors and encompassing education and dialogue on food security and the right to food;
2. Access to Food, grounded in poverty reduction as well as access to safe and nutritious food, healthy eating practices, and traditional food acquisition by Aboriginal communities;
3. *Sustainable Agriculture and Rural Development*; including agricultural research and development to increase production, and mitigation/adaptation measures to address climate change;
4. *Trade and Food Security*, which envisions fair trade as essential to food security and that trade liberalization can have negative effects on particular population groups or nations;
5. *Emergency Prevention and Preparedness*, which focuses principally on global conflicts and food aid; and
6. *Promoting Investment* in processing and production capacity.

The seventh commitment, *Implementation and Monitoring*, is only addressed in the international context. Of the ten priorities for action, the top two are the right to food and the reduction of poverty. Canada’s *Action Plan* goes on to describe measures to address the right to food, including applying a participatory approach to defining this core value.

Over the next ten years, Canada reported on its WFS commitments through a series of *Progress Reports* to the FAO [19–23]. The first *Progress Report* was issued in 1999 and four subsequent reports were released in 2002, 2004, 2006, and 2008. No further reports are anticipated.

The structure of the *Progress Reports* has changed over time. The first three reports reiterate the *Action Plan* priorities and offer an update on the seven commitments, with actions grouped under ‘domestic implementation’ and ‘international implementation’. The reports adopt a narrative form and initiatives are grouped under the specific commitments they address. The format of the fourth and fifth reports change. They continue to list the seven commitments, but there is no mention of the original priorities. Implementation updates are converted to tables. By the fifth report, the implementation tables have been substantially abbreviated and do not link to the seven commitments at all.

Some report sections are conspicuously static. For example, ‘Lessons Learned’ vary little between reports three and five. The section on Aboriginal peoples’ experience of food insecurity is virtually reproduced from report to report. This is concerning given the severity of food insecurity and challenges for healthy diets among Aboriginal communities expressed each time, attributed to income, but also to food quality and access, relating to the interface between traditional and contemporary ways of life [28, 29].

### The dynamics of frame conflict on food insecurity

#### Frame shifts: right to food and health

One of the most obvious patterns in the dynamics of framing and reframing across Canada’s WFS reports is change over time. This aligns with the longitudinal political context, in which Canada experienced a notable political shift. The Liberal Party, incumbent with a strong majority since 1993, changed its leadership in 2003 and was reduced to a minority government in the 2004 general election. The Conservative Party successfully united a centre-right and right wing party to win a minority in the 2006 general election and a majority in 2011.

Two prime examples of frame shifts are how the *Progress Reports* handle the right to food and how health is contextualized. The right to food was central to the WFS and Canada’s *Action Plan*. Canada’s early *Progress Reports* reiterate the right to food as a priority. By the third report, Canada is named as the “developed nation” example among case studies commissioned by FAO on the right to food, offering “important recommendations and lessons learned for federal, provincial, territorial and local governments to improve food security in Canada” ([21], p19). Remarkably, the next two reports eliminate mention of the right to food altogether, and only discuss human rights in the context of international development, foreshadowing the UN Special Rapporteur’s clash with the federal government in 2013.

Health conditions associated with food insecurity are another frame shift. The *Action Plan* notes that food insecurity is “compounded by difficulties in accessing appropriate social services, particularly among the aged and people with physical and mental disabilities, or with acute or chronic illness” ([3], p14); chronic disease amplifies the effects of inadequate household income. By the fourth and fifth *Progress Reports*, chronic disease becomes a food insecurity issue requiring attention. Obesity, “one of the leading causes of chronic illness in Canada… has focused attention on a whole new set of food security issues related to food quality and diet” ([22], p6). Prevention and control strategies such as Canada’s Diabetes Strategy ([22] p17) and the federal Aboriginal Diabetes Initiative ([23], p16) emerge as part of the food security implementation plan. The fourth report names the Diabetes Strategy under *Action Plan* commitment two, “policies aimed at eradicating poverty and inequality and improving physical and economic access by all, at all times, to sufficient, nutritionally adequate and safe food and its effective utilization,” alongside income-support measures such as the National Child Benefit ([22], p15).

#### Frame blending: poverty and complexity

We observed a second pattern of reframing through frame dilution, a version of what Rein and Schön have called frame “blending” ([18], p101). Blending occurs when policy actors need to cope in a pragmatic way with conflict. Rather than jettison old frames for new ones, actors blend aspects of the old and new frames into something workable and legitimate in the new context. Our term ‘dilution’ demonstrates how it is not only the admixture of frames that constructs the issue. Rather, strong frames can be qualified, toned down, and hence diluted.

The second *Progress Report*, for example, cites among its ‘Lessons Learned’ that, “There is a tendency among development practitioners to believe that programs aimed at reducing poverty will reduce food security. While poverty is unquestionably a major contributor to food insecurity, not all the poor are hungry” ([20], p59). This framing portrays food insecurity as only a narrow concern, in contrast to a broad societal concern deserving of substantive collective action, i.e., social policy interventions. The *Progress Reports* go on to feature targeted policy instruments for those most vulnerable, such as the National Child Benefit, a household income supplement for families with children who would benefit from workforce attachment, and the federal Food Mail Program (now Nutrition North Canada), a retailer/supplier subsidy for healthier perishable foods in northern communities facing high retail food prices. The only instance where one of the *Progress Reports* identifies a comprehensive poverty reduction approach is to name a provincial (Newfoundland and Labrador), and not a national, strategy ([22], p23). Canada’s federal governance structure tends to highlight social assistance as a provincial responsibility, but this is also an indicator of where proposed policy solutions reflect a dilution of the problem of poverty in relation to food security. Canada’s original *Action Plan*, in contrast, highlighted the “exceptional paradox” in the global co-existence of ample food production and food insecurity, naming poverty reduction as a priority area ([3], p4).

A second example of dilution is the evoking of complexity, which, from a frame-critical perspective, can represent a reframing of policy solutions without clear alignments to actors empowered to take action. The first *Progress Report* notes that “Efforts have to be made for all players to seek new and more creative partnerships, strengthen networks, and work inclusively, while encouraging participation of communities and individuals in developing and implementing policies and programs” ([19], p41). The second report proceeds, “Appropriate legislative and policy initiatives to address the problem of hunger can be undertaken *only* by the appropriate level of government and the private sector [*emphasis added*]” ([20], p11). By the fourth report, complexity is an “outstanding lesson learned” in which “the issues relating to food security—from poverty alleviation to micro-nutrient enhancement—are universally complex and require long term commitments from all stakeholders for resolution and impact” ([22], p13).

Does ‘complexity’ thus reflect commitment to wider dialogue? The original *Action Plan* had called for intersectoral action to define the right to food. The fourth and fifth *Progress Reports* exclude mention of the right to food altogether, while recounting consultative actions focused on community food security (community-based food organizations, food security networks, and local food justice initiatives). We agree with the first *Progress Report* that “Broad community involvement has proven to be the most effective way to shift community norms” ([19], p42), in terms of the need for diverse actors in a society to have the opportunity to participate in shaping the policy discourse. However, the portrayal of community actions in the absence of a clear linkage to discourse on how the state can and should operationalize the right to food, has served to make the connection between community-level and systems-level, macro-social policy dialogue more ambiguous.

#### Within-frame conflict: monitoring across jurisdictions

Like other grey literature of this type, the *Progress Reports* emphasize ‘ongoing monitoring’ of the problem at hand, a frame expressing progress and accountability. For example, the following list of measures are varied monitoring actions named in the *Progress Reports*:

- Nutritious Food Basket (Health Canada) ([19], p22; [21], p23; [22], p16)
- Canadian Community Health Survey (CCHS) direct measure of prevalence of household food insecurity ([19], p23; [20], p33; [22], p19)
- Local research projects on food availability, food security, and nutritional vulnerability ([19], p23)
- Food consumption and nutrient intake surveys ([19], p23)
- Audits of existing social programs ([21], p20)
- Development of health indicators ([19], p22; [22], p19)
- Specific tools for vulnerable populations such as Northern communities and pregnant women ([19], p23; [21], p25; [23], p18)
- Sustainable food costing ([22], p22)
- Documentation of local community food security initiatives ([22], p21)

The reports’ framing of monitoring illustrates within-frame conflict, an incongruence between rhetoric and action [10]. Over time, the *Progress Reports* reflect few systematic or consensual approaches to documenting the problem of food insecurity across jurisdictions and levels of government. The only clear cross-jurisdictional monitoring instruments are the Nutritious Food Basket and the Household Food Security Survey Module, and local initiatives are represented within the scope of federal responses. The disparate array of measurement approaches presented is incongruous with the framing of rigour and consistency. Reports three to five define “The Need for Data” as “additional quantifiable information” and a ‘Lesson Learned’ [21–23].

## Discussion

Our analysis begins to unpack the rhetorical basis for policy conflict on food insecurity in Canada, as illustrated in the series of reports associated with Canada’s response to the 1996 World Food Summit. We have described the disappearance of the concept of the right to food from the policy discourse; a static portrayal of food insecurity among Aboriginal populations; the emergence of chronic disease as a problem of policy significance; the use of complexity framing alongside a diminishment of the importance of interventions to address income security and poverty; and an emphasis on monitoring that increasingly reports on small local initiatives without reference to how they link to a broader national conversation. Each of these findings could reflect idiosyncrasies in report production, but together, and in a significant and institutionally sanctioned document set, suggests that unresolved policy tensions exist in the framing of food insecurity in Canada.

We in no way wish to suggest that our frame-critical analysis of the WFS reports explains the debates about food insecurity in Canada today. What we wish to convey through our illustrative example of Canada’s WFS reports is that problem definition in the form of framing is selective – it rules in at the same time as it rules out. Frames and framing can construct policy decisions even before a decision per se has been made. Reflecting on Canada’s WFS Progress Reports, the solutions presented as addressing household food insecurity are largely those *already in existence*, which are then framed in terms of what dimension of need they address. It should be emphasized that such frames not only *represent* but can actually play a discursive role in *shaping* the likelihood—or not—of food insecurity rising in importance on the federal policy agenda in the future.

During the UN Special Rapporteur’s visit, De Schutter’s statements and the government’s responses highlighted a gap between what can be documented to be a problem, and what can be identified a problem that needs a solution. Food insecurity is not only a population level health condition that can be measured through epidemiological assessment, but something that can construed by and constructed by relevant actors to be a policy problem: i.e., someone’s responsibility, and a matter for state intervention—or not.

One way of characterizing public health policy conflicts is to document how they reflect concrete disagreements about the means and ends of policy. Frame analysis can help us to broaden our understanding of such conflicts to focus upon how actors actively negotiate policy meaning through the language of a debate [30]. When we examine conflicts through a frame-critical lens, we need to examine both what the debate is about as well as how the debate is created and reproduced in its social context: through time, in institutional texts, and in other forums where argumentation can be observed. Through the brief exploratory examples in this paper, we have highlighted how frame-critical analyses are essential for dissecting *how* policy can become ‘stuck’ in the process of change.

## Conclusion

The process of problematization through framing is a gateway to agenda-setting, establishing new policies, reworking old ones, or even making strategic decisions *not* to act (famously described by Bachrach and Baratz [31]). Because frames embed meaning, they have an institutional foundation, which is to say that they are ‘sponsored’, to use Schön and Rein’s term, through social structures and can thus be reconstructed through an analysis of policy documentation of different kinds, such as we have done in this manuscript. We thus contend that framing analysis should be one of the core steps in analysis and evaluation of public health policy issues and to form the basis of reframing towards new policy solutions.
